# Breastfeeding practices within the first six months of age in mid-western and eastern regions of Nepal: a health facility-based cross-sectional study

**DOI:** 10.1186/s12884-020-2754-0

**Published:** 2020-01-30

**Authors:** Dinesh Dharel, Ranjan Dhungana, Sushma Basnet, Swotantra Gautam, Animesh Dhungana, Rajesh Dudani, Asmita Bhattarai

**Affiliations:** 1 Department of Pediatrics, Cumming School of Medicine, University of Calgary, 1403 29th Street NW, Calgary, AB T2N 2T9 Canada; 2 BP Koirala Institute of Health Sciences, Dharan, Nepal; 3 Sushma Koirala Memorial Trust, Kathmandu, Nepal; 4 Lifeline Hospital Institute of Health Sciences, Damak, Jhapa Nepal; 5 Population Health Research Institute (PHRI), Hamilton, Canada; 6 Singapore Gorkha Hospital, Lalitpur, Nepal; 7 John H Stroger Hospital of Cook Country, Chicago, USA; 8 Department of Community Health Sciences, Cumming School of Medicine, University of Calgary, Calgary, Alberta Canada

**Keywords:** Breastfeeding: early, Exclusive, Predominant, Partial

## Abstract

**Background:**

The World Health Organization recommends initiation of breastfeeding within 1 hour of birth and exclusive breastfeeding up to 6 months of age. Infant feeding practices, including suboptimal breastfeeding practices, are associated with stunting. Rate of stunting was highest in the Mid-western region and lowest in the Eastern region of Nepal. This study aimed to assess the breastfeeding practices in these two regions, as well as to identify factors associated with partial breastfeeding.

**Methods:**

We conducted a health facility-based cross-sectional study in the Mid-western and Eastern regions of Nepal from December 2017 to May 2018. Investigators administered a pre-tested questionnaire among consecutive 574 mother-infant dyads at different levels of health facilities. We dichotomized the breastfeeding pattern to partial breastfeeding and full (exclusive or predominant) breastfeeding. We conducted multivariable logistic regression to identify factors associated with partial breastfeeding within 6 months of age.

**Results:**

There were 574 infants included in the study, all of which received at least some breastfeeding. Only 23.2% of infants were exclusively breastfed until 6 months, with 28.2% predominantly breastfed and 48.6% partially breastfed. Partial breastfeeding rate was 52.3% in the Mid-western region and 44.4% in the Eastern region. Breastfeeding was initiated within an hour from birth in 67.2% of infants. One-quarter of infants were given pre-lacteal feed, honey being the commonest. Knowledge of the recommended duration of exclusive breastfeeding was inadequate in 16, and 65% of mothers reported breastfeeding problems in the first 6 months. Firstborn and low birth weight infants had a significantly higher rate of partial breastfeeding. Partial breastfeeding was also higher when infants were not breastfed within 1 hour from birth, mothers reported having breastfeeding-related problems or had inadequate knowledge of the duration of exclusive breastfeeding.

**Conclusion:**

Nearly half of the infants were fully breastfed at 6 months of age in Nepal. The rate of partial breastfeeding was higher with inadequate knowledge on duration of exclusive breastfeeding or late initiation of breastfeeding or perceived breastfeeding problems. Hence, programs should address knowledge and practice gaps in breastfeeding practices, particularly among mothers of low birth weight and firstborn infants.

## Introduction

Malnutrition is a serious factor contributing to poor child health status. Under-nutrition, which includes sub-optimal breastfeeding, was responsible for 45% (3.1 million of 6.9 million) of under-five child deaths in 2011 globally. Nepal is one of the thirty-four countries that constitutes 90% of the global burden of malnutrition in children [1]. Nepal Demographic and Health Survey (NDHS) in 2016 reported that 36% of children are stunted (defined as height-for-age < − 2 z score), 10% are wasted (defined as weight-for-height < − 2 z score), and 27% are underweight (defines as weight-for-age < − 2 z score); based on the WHO growth chart) [2].

Stunting is a sign of chronic under-nutrition. In Nepal, stunting has a significant regional variation. Stunting is highest (42%) in the Mid-western region and lowest (32.6%) in the Eastern region [2]. The sustainable development goal target is to reduce underweight from 24.64 to 10.09% and stunting from 30.58 to 12.38% in under-five children from 2017 to 2025 in Nepal [3].

The World Health Organization has classified breastfeeding as exclusive, predominant, or complimentary [4]. Exclusive breastfeeding refers to the use of breast milk only (including expressed milk or milk from a wet nurse). It can include the use of medicine drops and syrups or oral rehydration solution. Predominant breastfeeding refers to breast milk used as a predominant source of nourishment. It can include water and water-based drinks, fruit juice, ritual fluids, and medicines. Complementary feeding refers to breast milk use along with any food or liquid, including non-human milk and formula. The interagency group for action on breastfeeding categorized breastfeeding into full or partial [5]. Full breastfeeding included exclusive or almost exclusive (predominant) breastfeeding, while partial breastfeeding reflected complementary feeding with high, medium, or low use of breast milk.

The WHO recommends exclusive breastfeeding up to 6 months of age, with continued breastfeeding along with appropriate complementary feeds up to 2 years of age or beyond [6]. The complementary feeding should be initiated in a child, no later than 26 weeks and not before 17 weeks of age [7]. The benefits of exclusive breastfeeding and proper weaning in the growth, development, and prevention of illness in young children are undisputable. The frequency, timing, and duration of breastfeeding, as well as the frequency, type, and amount of complementary feeding, have been crucial aspects of infant feeding practice. Better breastfeeding practices were shown to have a significant impact on the survival of infants in a limited resource setting of southern Nepal [8]. Moreover, feeding honey as pre-lacteal feed and animal milk or formula as a substitute for human milk pose a risk to these vulnerable infants. These other feeds might increase the risk of infection in the infants; by damaging the immature gut wall of the infants and making it more susceptible to infection transmission. There is also an increased risk of infection if the water used to prepare the feed is unsafe, particularly in low resource settings [9]. As per Nepal Demographic Health Survey 2016, infant and young child feeding practices are sub-optimal, with about 65% exclusive breastfed under 6 months and about 55% colostrum fed within an hour of birth. Only 41% of children aged 4–5 months were exclusively breastfed compared to 80% in 0–1 months and 72% in 2–3 months. However, all infants had received at least some breast milk by 4–5 months of age. In addition to breast milk, 13.7% received water, 4.7% received non-milk liquid, 11.5% received non-human milk or formula milk, and 29.2% received other semi-solid or solid feed [2]. Prelacteal feeding, withholding of colostrum, and being a first-time mother were associated with a significantly higher rate of partial breastfeeding in a study done in eight countries, including Nepal [10]. Thus, there is a room for improvement in reducing partial breastfeeding and sustaining exclusive breastfeeding until the recommended 6 months of age. And, this needs a better understanding of breastfeeding pattern and determinants of partial breastfeeding in the context of Nepal.

Hence this study was conducted to assess the breastfeeding practices in the eastern and mid-western regions of Nepal. We aimed to determine full (exclusive or predominant) and partial breastfeeding rates in these two regions. We also aimed to identify factors associated with partial breastfeeding in Nepal.

## Methods

### Study setting

We conducted this study in two regions of Nepal: the Eastern region that had the lowest prevalence of stunting and the Mid-western region that had the highest prevalence of stunting. We chose the health facilities with immunization and outpatient clinics, as these are the places where we could conveniently find most mothers with a child aged 6 to 18 months. The study sites were chosen conveniently. The health facilities included one hospital in Dhankuta district and two hospitals, one primary health care center, and one health post from the Jhapa district in the Eastern region and one hospital in Banke district in the Mid-western region. The study sites were Bheri Zonal Hospital in Banke, District Hospital in Dhankuta, and Damak Hospital, Lifeline Hospital, Gaurigunj Primary Health Care Centre, and Rajgadh Health Post in Jhapa.

### Study design, duration, and participants

The design of the study was a health facility-based cross-sectional study. We collected data over 6 month’s period (December 2017 to May 2018). Mother of 6 to 18 months old child was contacted for participation in the study, when they finished their appointment in the outpatient clinic of the health facility for immunization or minor ailments of the child. We excluded if the child was ill or parent was not interested in participating. The study sampled mothers of children aged 6 to 18 months because this will allow us to ask all the mothers about the history of breastfeeding patterns within the first 6 months of age. We included infants till the age of 18 months to ensure that we get enough sample size. We calculated the sample size from Open Source Epidemiologic Statistics for Public Health [11]; with 65% national prevalence of exclusive breastfeeding within 6 months of age reported by NDHS 2016 [2], and the desired confidence interval of 95 and 20% absolute precision [12]. We aimed for 10% oversampling from calculated 568 and finally had a total of 574 participants completing the questionnaire.

### Instrument and data collection

One of the investigators conducted face-to-face interviews with the mother using a pre-tested structured questionnaire. The questionnaire was adapted from the Nepal Demographic and Health Survey 2016 and updated based on variables included in a similar study done in Bhaktapur, Nepal [2, 13], We pre-tested the Nepali version of the questionnaire to ensure lingual and cultural appropriateness before use in this study. Pre-testing was done among 30 eligible participants in another district (Sunsari) with similar demographic characteristics. The questionnaire included bio-demographic information of mother and child. It focused on the practice of breastfeeding during the first 6 months of age. The questionnaire was read aloud to the participants by the investigator and check-marked in the appropriate responses. The investigator noted any additional information or feeding option offered by the participant during the interview.

### Variables

We followed WHO guidelines to describe the breastfeeding pattern [4]. We focused on a period of 6 months since birth [5]. Exclusive breastfeeding was defined as the infant receiving milk from the mother or a wet nurse with no other food except syrup medicine. Predominant breastfeeding was defined as the infant receiving breast milk only as a source of nutrition and received water or water-based drinks like tea or local herbal drops. Partial breastfeeding was defined as the infant receiving non-human milk feeds such as animal milk, formula milk, vegetable soup, lentil, or other solid or semisolid food. The primary outcome variable, assessing breastfeeding pattern, was dichotomized as full (exclusive or predominant) and partial breastfeeding.

Predictor demographic variables included age, education, and occupation of the mother, socioeconomic status, religion, caste stratification, type of family, birth order, gender, birth weight, gestational age, mode of delivery and place of delivery of the infant. Variables related to breastfeeding practices included the time of initiation of breastfeeding, colostrum feeding, pre-lacteal feeding, self-reported breastfeeding problems, and knowledge of the recommended duration of exclusive breastfeeding.

We categorized caste stratification as highly disadvantaged, disadvantaged, and relatively advantaged [14]. As there were fewer observations in the relatively advantaged category, we collapsed disadvantaged/relatively advantaged (< 40% of people below the poverty line) and highly disadvantaged (> 40% of people below the poverty line). Socioeconomic status was categorized using the Kupuswamy scale modified in the context of Nepal, which gives a score to education, occupation, and income of households to classify on a 1 to 5 ordinal scale [15]. Similarly, since there were fewer observations in the lowest and highest score categories, we collapsed the categories into three (lower, middle, and upper score). We categorized the mother’s occupation as a homemaker or working mother (including any paid work outside of the home). Birth weight was recorded as ‘low’ (< 2500 g) and ‘normal’ (≥2500 g). Gestational age was recorded as preterm (< 37 weeks) or term (37 weeks or more).

Initiation of breastfeeding was categorized as early if breast milk was given within 1 hour from birth; any time after 1 hour was categorized as late. Pre-lacteal feeding was defined as any feed or drink given before breastfeeding was initiated. We categorized knowledge of the recommended duration of breastfeeding as correct if the response was up to 6 months and any other or no answer to the open-ended question as incorrect. Self-reported breastfeeding problems included any one of perceived inadequate breast milk secretion, engorged breast, crack nipple, or inverted nipple. These occurred at any time in the first 6 months of infancy.

### Statistical analysis

We presented the results as mean with standard deviation, frequencies, and proportions, as appropriate. We showed them in tables and bar diagrams. The descriptive statistics were stratified according to the regions and breastfeeding patterns. We conducted the univariate logistic regression between all pre-identified variables with breastfeeding patterns as partial breastfeeding versus full breastfeeding. The factors which were significantly associated with partial breastfeeding (*p*-value < 0.1) were subjected to multivariable logistic regression, using a stepwise backward Wald method. The unadjusted and adjusted odds ratios, along with their 95% confidence interval, have been reported. We did all analyses using the Statistical Package for Social Sciences (SPSS, IBM Statistics, Version 16.0).

### Ethics approval and consent

We obtained ethical approval from the Nepal Health Research Council (NHRC proposal ID 3742017). We also received permission for data collection from the respective District Public Health Offices. Participants provided informed consent for themselves and their infants.

## Results

### Characteristics of participants

The questionnaire was completed by 574 mother-infant dyads, with 306 (53.3%) being from the mid-western region and 268 from the eastern region; this number constituted 91.7% of 626 mothers who were recruited for participation in the study, excluding those with incomplete data. The mean age of mothers was 25.38 ± 4.14 years, and that of infants was 9.98 ± 2.26 months. The median size of their family was 5 (IQR 4–7), and there was a median of 1 (IQR 1–2) child under 5 years of age. Table 1 illustrates the demographic characteristics of the participants, stratified by region, and breastfeeding pattern (partial or full). Maternal education, working status of the mother, caste stratification based on below poverty level, and socioeconomic status based on the modified Kupuswamy scale were significantly different between the eastern and mid-western regions.

**Table 1:** Background characteristics of population by region and breastfeeding status (*N* = 574)

Factor	Category	Eastern region *n* = 268		Mid-western region *n* = 306		*P* value
		Partial breastfeeding N (%)	Full breastfeeding N (%)	Partial breastfeeding N (%)	Full breastfeeding N (%)	
Age of mother	< 20 years	7 (5.9)	5 (3.4)	5 (3.1)	10 (6.8)	0.811
	> 20 years	112 (94.1)	144 (96.6)	155 (96.9)	136 (93.2)	
Maternal education	No formal education	5 (4.2)	8 (5.4)	27 (16.9)	23 (15.8)	< 0.001
	School level education	70 (58.8)	74 (49.7)	116 (72.5)	99 (67.8)	
	College level education	44 (37)	67 (45)	17 (10.6)	24 (16.4)	
Occupation of mother	Homemaker mother	84 (70.6)	80 (43.7)	127 (79.4)	115 (78.8)	< 0.001
	Working mother	35 (29.4)	69 (46.3)	33 (20.6)	31 (21.3)	
Urbanization	Rural municipality	62 (52.1)	91 (61.1)	101 (63.1)	96 (65.8)	0.074
	Urban municipality	57 (47.9)	58 (38.9)	59 (36.9)	50 (34.2)	
Caste stratification (based on BPL cutoff)	Highly disadvantaged (> 40% BPL)	3 (2.5)	15 (10.1)	22 (13.8)	16 (11)	0.022
	Disadvantaged/relatively advantaged (<=40% BPL)	116 (97.5)	149 (89.9)	138 (86.2)	130 (89)	
Socio-economic status	Middle/high score 11–29	84 (70.6)	112 (75.2)	148 (92.5)	126 (86.3)	< 0.001
	lower score 0–10	35 (29.4)	37 (24.8)	12 (7.5)	20 (13.7)	

### Breastfeeding practices

**Fig. 1:**
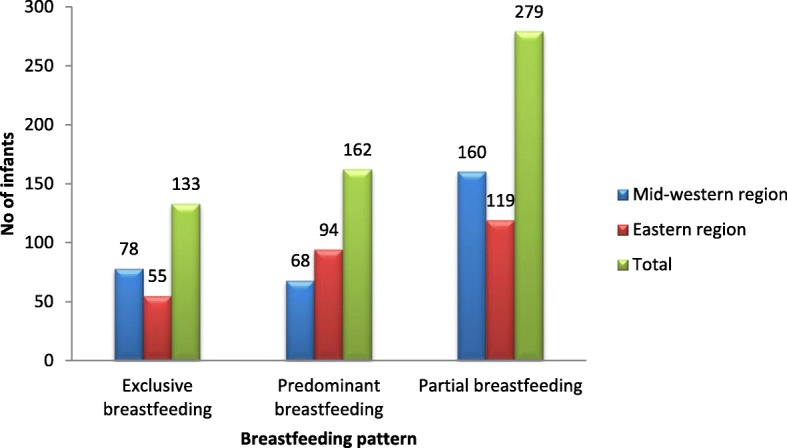
Pattern of breastfeeding practice in Mid-western and Eastern Regions of Nepal. First blue bar: Mid-western region. Second red bar: Eastern region. Third green bar: Total

Out of the total 574 children, only 23.2% (*n* = 133) were exclusively breastfed, and 28.2% (*n* = 162) were predominantly breastfed until the age of 6 months. Nearly half, 48.6% (*n* = 279) infants were partially breastfed for the first 6 months of their age. All infants in the study had received breastfeeding at some time in the first 6 months of age. Partial breastfeeding rate was higher (52.3%) in the Mid-western region compared to 44.4% in the Eastern region. Figure 1 illustrates the breastfeeding pattern disintegrated by region.

Breastfeeding was initiated within an hour of birth in 67.2% of infants, but 8.2% of infants were not breastfed until 24 h after delivery. Eighty-four percent of mothers knew that the recommended age for exclusive breastfeeding was 6 months. Only 2.3% of infants did not receive colostrum, but one quarter (141/574) of the infants were given pre-lacteal feeds. Honey was the most common (13.6%) pre-lacteal feed given to infants, followed by formula milk (8%) and animal milk (2.3%). One fifth (113/574) of mothers perceived that their milk secretion was not adequate for their baby. Nearly two-thirds of the mothers (64.6%) reported having at least one breastfeeding problem at some point in the first 6 months of age (one of: perceived inadequate secretion of breast milk, engorged breasts, inverted nipple, or cracked nipple). Breastfeeding variables stratified by region and breastfeeding pattern are shown in Table 2. There was a significant difference in these variables between the eastern and mid-western regions, except for full or partial breastfeeding, which did not reach the level of significance (*p*-value 0.059).

**Table 2:** Prevalence of breastfeeding related variables stratified by region and breastfeeding status

Breastfeeding related variables	Category	Eastern region N (%)	Mid-western region N (%)	Total N (%)	*P*-value
Breastfeeding pattern	Full	149 (55.6)	146 (47.4)	295 (51.4)	0.059
	Partial	119 (44.4)	160 (52.3)	279 (48.6)	
Initiation of breastfeeding	Within one hour	155 (57.8)	231 (75.5)	386 (67.2)	< 0.001
	After one hour	113 (42.5)	75 (24.5)	188 (32.8)	
Colostrum feeding	No	2 (0.7)	11 (3.6)	13 (2.3)	0.022
	Yes	266 (99.3)	295 (96.4)	561 (97.7)	
Pre-lacteal feeding	No	155 (57.8)	278 (90.8)	433 (75.4)	< 0.001
	Yes	113 (42.2)	28 (9.2)	141 (24.6)	
Self-reported breastfeeding problems	Yes	200 (74.6)	171 (55.9)	371 (64.6)	< 0.001
	No	68 (25.4)	135 (44.1)	203 (35.4)	
Knowledge about exclusive breastfeeding duration	Incorrect response	13 (4.9)	79 (25.8)	92 (16)	< 0.001
	Correct response	255 (95.1)	227 (74.2)	482 (84)	

The mean age of starting complementary feeding (any formula or animal milk feeding or semisolid/solid feed) was 5.06 ± 1.74 months. Complementary feeding was started before 5 months of age in 22.5% (*n* = 129) of infants. Formula milk was given in 79% (*n* = 102) and animal milk in 29% (*n* = 65). Breastfeeding was continued despite the initiation of complementary feeding.

### Factors associated with breastfeeding practices

On univariate logistic regression, maternal education, working status of the mother, birth order, birth weight, early initiation of breastfeeding within 1 hour of birth, colostrum feeding, self-reported breastfeeding problems, knowledge of the appropriate duration of breastfeeding, and developmental region were significantly associated with partial breastfeeding (*p*-value < 0.05). Partial breastfeeding rate was higher in the mid-western region compared to the eastern region, but it did not reach the level of significance (p-value = 0.06). Multivariable logistic regression, including the variables as mentioned earlier in the model, showed that firstborn and low birth weight infants independently predicted higher rates of partial breastfeeding. Other independent predictors were the initiation of breastfeeding after 1 hour from birth, inadequate knowledge of the duration of exclusive breastfeeding, and perceived breastfeeding problems in the first 6 months, as shown in Table 3.

**Table 3:** Association of factors associated with partial breastfeeding in Nepal

Predictor variables	Unadjusted OR (95% CI)	Adjusted OR (95% CI)
Birth Order		
Higher birth order	1.0	1.0
First birth order	−0.133 (−0.217, −0.052)	1.971 (*1.374,2.827)
Birth weight		
Low (< 2.5 kg)	1.0	1.0
Normal (≥ 2.5 kg)	0.139 (0.063, 0.239)	0.572* (0.388,0.845)
Occupation of mother		
Home maker mother	1.0	1.0
Working mother	0.102 (0.023,0.202)	0.706 (0.474,1.050)
Residence by region		
Mid-western region	1.0	1.0
Eastern region	0.079 (−0.003, 0.161)	0.701 (0.473,1.041)
Knowledge of duration of exclusive breastfeeding		
Incorrect response	1.0	1.0
Correct response	0.155 (0.100, 0.321)	0.471* (0.282,0.787)
Initiation of breastfeeding		
After one hour from birth	1.0	
Within an hour of birth	0.101 (0.021, 0.195)	0.582* (0.398,0.851)
Breastfeeding problems		
Self-reported to be absent	1.0	
Self-reported to be present	−0.143 (−0.235, −0.065)	0.466* (0.320, 0.679)
Colostrum feeding		
Not Given	1.0	1.0
Given	0.086 (0.015, 0.565)	1.550 (0.398, 6.040)

Footnote: Variable(s) entered on step 1 of backward stepwise wald regression: Working Mother, Mid-western residence, First birth order, normal birth weight, knowledge of duration of breastfeeding, self-reported breastfeeding problem, initiation of breastfeeding within an hour, colostrum feeding

*Statistically significant

## Discussion

Our study was a questionnaire-based cross-sectional study conducted in two different regions of Nepal, among mother-infant dyad visiting health facilities for immunization or outpatient visit at 6 to 18 months of age. The results showed that the rate of exclusive breastfeeding until 6 months of age was 23.2%, and predominant breastfeeding was 28.2%. Nearly half (48.6%) of the infants received partial breastfeeding and no infant who did not receive any breastfeeding. The rate of partial breastfeeding was higher in the Mid-western region (52.3%) compared to the Eastern region (44.4%), but the difference was not statistically significant.

Compared to two-third of infants being exclusively breastfed as of NDHS 2016 [2], only 23.2% of infants were exclusively breastfed in this study population. However, the full breastfeeding rate, including predominant breastfeeding, was 51.4% when predominant breastfeeding is added. Bhandari et al. reported the rate of predominant breastfeeding, defined as mainly breastfed, not fed solid/semisolid foods, infant formula, or non-human milk to be 57.2% in Nepal [16]. The global exclusive breastfeeding rate currently stands at 40%, based on data from 129 countries. But, only 23 countries have exclusive breastfeeding rates above 60%, which is the new global target set to meet by 2030 [17]. The national or global average rates mask the dramatic disparities that exist in different regions and across countries. An interesting observation in our study was that all the infants received some breastfeeding within 6 months of age. One study from a southeastern state in America showed that 38% of mothers did not initiate breastfeeding at all [18]. There are some rare medical contraindications to breastfeeding. Still, the preference of mothers to breastfeeding can contribute to the huge fraction of infants who receive exclusive formula feeding. The socio-cultural aspect of high choice for breastfeeding in Nepal needs further exploration.

Studies have shown the crucial role of exclusive breastfeeding in the growth of children [19, 20]. However, this study did not establish the regions of residence, that had the highest and the lowest rates of stunting, as an independent predictor factor for the higher rate of partial breastfeeding. Still, other breastfeeding practices such as early initiation of breastfeeding within an hour from birth, colostrum feeding, pre-lacteal feeding were significantly different between the eastern and mid-western regions and the knowledge of the recommended duration of breastfeeding as well as perceived breastfeeding problems in the first 6 months of age were also significantly different. The two regions differed significantly on maternal education, working status of the mother, caste stratification based on below poverty level, and socioeconomic status based on the modified Kupuswamy scale. Further study would be needed to investigate if these breastfeeding-related variables would be contributory to the difference in the rate of stunting, after adjusting for other regional variations.

Breastfeeding was initiated within an hour from birth in 67.2% of infants in our study. This rate was similar to 67% observed by Karkee et al. in central Nepal [21] but more than the national average of 54.9% reported by NDHS 2016 [2]. and 41.8% reported by Bhandari et al. [16] Rate of early initiation of breastfeeding varied widely among countries, ranging from 17.7 to 98.4% with a mean of 57.6% [22].

A quarter of all infants in this study were given pre-lacteal feed, honey being the commonest one, followed by formula milk and then animal milk. However, a higher rate (30.6%) of pre-lacteal feeding was reported in the south-western region of Nepal. In their study, formula milk was the most common, followed by animal milk and then sugar water [23]. These rates were much higher (9.1%) than that reported in central Nepal [21] but comparable (29%) to that reported in NDHS 2016 [2]. Complementary feeding was started at a mean age of 5.06 ± 1.74 months in this study cohort, with a quarter of infants beginning at under 5 months of age. Formula or animal milk was most commonly used as a first complementary feed. The prevalence of formula feeding in western Nepal was 7.5% in the first month and 17% in the sixth month [24]. But, nearly half of infants 6–24 months were given formula or animal milk, as per NDHS 2016 [2].

Our study showed that infants who were born smaller than 2.5 kg and born as a first child have higher rates of partial breastfeeding within 6 months of age. Babies born small have more medical issues impairing their readiness and ability to initiate and continue breastfeeding compared to those born normal weight. Mothers of premature babies may not have resources to establish and maintain milk supply until their infant can breastfeed. Pre-lacteal feeding (in 4–63% of infants), withholding of colostrum (in 2–16% of infants), and being a first-time mother were significantly associated with partial breastfeeding among 2053 infants in the first month of life in eight developing countries [10]. Being a first-time mother was independently associated with the failure of exclusive breastfeeding in a Swedish cohort in which only 77% of mothers exclusively breastfed at 2 months postpartum [25].

Eighty-four percent of mothers participating in this study knew the recommended duration of exclusive breastfeeding. Nearly three-fourth of 639 lactating women in a hilly district of central Nepal had received information on breastfeeding [26]. The positive association of knowledge about exclusive breastfeeding with appropriate infant feeding practices is apparent. However, knowledge alone is not enough. The knowledge-practice gap in the duration of exclusive breastfeeding was demonstrated in a study done in Kathmandu, Nepal, with 87% showing good knowledge and only 33% practicing it [27].

Perceived problems with breastfeeding, particularly related to inadequate secretion of the milk, are not uncommon in Nepal. Nearly 85% of mothers perceived that their breast milk was sufficient at 4 weeks, but only 54.7% continued to feel so at 22 weeks postpartum in Kaski district [26]. In a study conducted in Bhaktapur district, 51.4% of mothers initiated other feeding methods, most commonly animal milk or formula milk, due to perceived inadequate milk secretion [13]. A qualitative study in a semi-urban area of Kathmandu supported such perception of inadequate milk secretion to be contributory to early initiation of complementary feeding [28]. Anxious mothers tend to perceive that they have inadequate secretion of breast milk attributing to non-specific and frequently inaccurate signs such as infant cry.

Several socio-demographic factors attributed by previous studies to affect the exclusive breastfeeding rate in developing countries, including Nepal, were found not to be significantly associated with our study. Maternal education and occupation were statistically significant in univariate regression analysis, but they lost lost significance when adjusted to other variables in multivariate logistic regression. Higher maternal education, relatively advantaged caste stratification, occupation of father, institutional delivery, and vaginal delivery, along with information and awareness about exclusive breastfeeding and practice of early colostrum feeding, were associated with exclusive breastfeeding in slum areas of Kathmandu, Nepal [29]. Higher maternal education, hospital delivery, receiving postnatal care, and possession of radio were associated with better infant and young child feeding practices in slum areas of Bahir Dar city in Ethiopia [30]. In a community-based study in Bhaktapur, Nepal, no school education of mother, religion other than Hindu, working mother, and joint family was shown to be associated with poorer breastfeeding practices [31]. However, in a health facility-based study conducted in Bhaktapur, Nepal, mother’s knowledge of the duration of exclusive breastfeeding and not living in joint families were shown to be associated with increased exclusive or predominant breastfeeding for four or more months [13].

### Limitations

This questionnaire-based cross-sectional study recruiting participants as mother-infant dyad visiting various levels of health facilities in two different regions of Nepal posed some issues in generalizing the findings of this study. Non-random sampling could lead to selection bias. Also, health facility is not an ideal setting for studying breastfeeding patterns and factors influencing exclusive breastfeeding at population level. We utilized the immunization clinics and outpatient departments of high-volume hospitals in study districts. We approached mothers who brought their young child for non-critical and non-urgent health visits. Selecting such a setting optimized their response in collecting data about their infant’s nutrition. Data collection was done through structured questionnaires by investigators not related to their health service, thus minimizing the risk of information bias.

A comparison of the rate of exclusive breastfeeding is often limited by the variation in the definition used. The preciseness of infants’ age in which complementary feeding was reported or documented in questionnaire-based studies also leads to potential limitations. This study used the standard definition recommended by WHO 2008 [4]. Although the category of predominantly breastfed infants reflected a less than optimal breastfeeding pattern compared to exclusively breastfed infants, both groups had human milk as a source of nutrition. Hence, full breastfeeding, which included exclusive and predominant breastfeeding, was compared with the partial breastfeeding group. However, these definitions do not encompass how the infant is fed, pumping or expressing breast milk and bottle feeding [32].

Feeding pattern of infants under 6 months of age was studied by asking the mother of 6 to 18 months old children. Hence, recall bias is possible. One study showed that the age of weaning was overestimated approximately 1 month in interviews 1–3.5 years after birth compared to those within 3 weeks of the event [33]. In addition, the data on breastfeeding problems was self-reported and could not be validated.

## Conclusion

Nearly half of the infants were fully (exclusively or predominantly) breastfed at 6 months of age in Nepal, with a slightly higher rate of partial breastfeeding in the Mid-western region compared to the Eastern region. The rate of partial breastfeeding was more among small and firstborn infants. It was also higher when mothers did not initiate early breastfeeding or perceived breastfeeding problems or did not know the appropriate duration of exclusive breastfeeding. Nutrition programs for infants should consider targeting mothers of low birth weight and firstborn infants and focus on improving the knowledge and practice of early and exclusive breastfeeding.

## Data Availability

The datasets used and/or analyzed during the current study are available from the corresponding author on reasonable request.

## Abbreviations

BEOC: Basic Emergency Obstetric Care
BPL: Below Poverty Level
CEOC: Comprehensive Emergency Obstetric Care
CI: Confidence Interval
IQR: Interquartile Range
NDHS: Nepal Demographic Health Survey
NHRC: Nepal Health Research Council
WHO: World Health Organisation
